# Leg elevation decreases the incidence of post-spinal hypotension in cesarean section: a randomized controlled trial

**DOI:** 10.1186/s12871-017-0349-8

**Published:** 2017-04-24

**Authors:** Ahmed Hasanin, Ahmed Aiyad, Ahmed Elsakka, Atef Kamel, Reham Fouad, Mohamed Osman, Ali Mokhtar, Sherin Refaat, Yasmin Hassabelnaby

**Affiliations:** 1 Department of Anesthesia and Critical Care Medicine, Cairo University, Cairo, Egypt; 2 Department of Obstetrics and Gynecology, Cairo University, Cairo, Egypt

**Keywords:** Hypotension, Spinal anesthesia, Cesarean section, Leg elevation

## Abstract

**Background:**

Maternal hypotension is a common complication after spinal anesthesia for cesarean section (CS). In this study we investigated the role of leg elevation (LE) as a method for prevention of post-spinal hypotension (PSH) for cesarean section.

**Methods:**

One hundred and fifty full term parturients scheduled for CS were included in the study. Patients were randomized into two groups: Group LE (leg elevation group, *n* = 75) and group C (Control group, *n* = 75). Spinal block was performed in sitting position after administration of 10 mL/Kg Ringer’s lactate as fluid preload. After successful intrathecal injection of local anesthetic, Patients were positioned in the supine position. Leg elevation was performed for LE group directly after spinal anesthesia and maintained till skin incision. Intraoperative hemodynamic parameters (Arterial blood pressure and heart rate), intra-operative ephedrine consumption, incidence of PSH, and incidence of nausea and vomiting were reported.

**Results:**

LE group showed lower incidence of PSH (34.7% Vs 58.7%, *P* = 0.005) compared to the control group. Arterial blood pressure was higher in the LE group compared to the control group in the first two readings after spinal block. Other readings showed comparable arterial blood pressure and heart rate values between both study groups; however, LE showed less ephedrine consumption (4.9 ± 7.8 mg Vs 10 ± 11 mg, *P* = 0.001).

**Conclusion:**

LE performed immediately after spinal block reduced the incidence of PSH in parturients undergoing CS.

**Trial registration:**

The study was registered at Pan African Clinical Trials Registry system on 5/10/2015 with trial number PACTR201510001295348.

## Background

Maternal hypotension is common after spinal anesthesia for Cesarean section (CS) with an incidence up to (60–70%) [1, 2]. Post-spinal hypotension (PSH) in Cesarean section has been associated with many maternal and fetal complications [1]. PSH is mainly due to decreased vascular tone leading to decreased systemic vascular resistance and decreased venous return [2]. Thus, measures used for prevention and management of PSH are mainly concerned by increasing vascular tone and increasing venous return which can be achieved by using vasopressors, fluid administration, and positioning regimens [1–4]. Although many measures have been reported for prevention and management of PSH [3, 4], none of these measures totally eliminated the occurrence of hypotension [3].

Leg elevation (LE) creates an increase in venous return by translocation of blood from lower extremities to the thorax. Thus, LE leads to increased stroke volume (SV) and consequently cardiac output (CO) [5]. LE was previously used as a first aid maneuver in acute circulatory collapse [6], it has been also considered as a popular method for detection of fluid responsiveness [7]. The evidence for a possible role for LE in prevention of PSH is unclear. In this study, we investigated the efficacy of LE performed after spinal block in prophylaxis of PSH during CS.

## Methods

This randomized controlled study was conducted at Cairo university hospitals after institutional research committee approval (N-47-2015). The study was registered at Pan African Clinical Trials Registry system on 5/10/2015 with trial number PACTR201510001295348. The study was performed during the period starting from January 2016 till May 2016. Written informed consents were obtained from participants before inclusion. Patients were randomly divided using computer generated sequence into two groups: LE group (*n* = 75) and control group (*n* = 75) with the use of opaque sealed envelopes.

Participants were full term (between 39 weeks and 40 weeks 6 days) singleton parturient aged between 18 and 38 years scheduled for CS under spinal block. All patients were required to be American Society of anesthesiologists (ASA) physical status I/II. Exclusion criteria were cardiovascular diseases, hypertensive disorders of pregnancy, and body mass index (BMI) above 30 kg/m^2^. Patients with baseline hypotension (systolic blood pressure (SBP) < 100 mmHg) or antepartum bleeding were also excluded from the study.

Before spinal block, all patients received 10 ml/kg Ringer’s lactate as a preload via an 18-G intravenous catheter and were pre-medicated with metoclopramide (10 mg intravenous), ranitidine (50 mg intravenous) and dexamethasone (8 mg intravenous). Spinal block was performed in the sitting position at L3-4 or L4-5 interspace. A 25-G spinal needle was used for intrathecal injection of 10 mg hyperbaric bupivacaine in addition to 25 μg fentanyl. Monitors included pulse oximetry, non-invasive blood pressure monitor, and electrocardiograph (ECG).

Immediately after spinal block, patients were positioned in supine position without left uterine displacement and divided into two groups:

- LE group: leg elevation for 30 cm using two standard pillows positioned under the heels (so that the leg is elevated approximately 40° above the horizontal plane) till skin incision
- Control group: regular supine position

Sensory level was checked to be at T4 by pinprick. Any case with sensory level below T4 was considered as “failed spinal block” and excluded from the study. At skin incision, Patients in both study groups were placed in supine un-wedged position. Monitors for vital signs (Non-invasive blood pressure monitor, pulse oximetry, and ECG) were applied prior to spinal block till the end of the operation. Hypotension (defined as either SBP < 90 mmHg or decreased SBP by 25% from the baseline reading) was managed using ephedrine increments (3 mg). Bradycardia (defined as heart rate below 60 bpm) was managed by atropine (0.5 mg). After delivery of the fetus, intravenous oxytocin infusion was given (20 IU/L at the rate of 120 mL/h).

Our primary outcome was incidence of PSH (defined as the percent of parturients who showed at least one episode of hypotension during the period starting from spinal block till delivery of the fetus). Secondary outcome parameters included the incidence of bradycardia (defined as the percent of parturients who showed at least one episode of bradycardia during the period starting from spinal block till delivery of the fetus), total ephedrine requirements, number of hypotensive episodes, urine output, and blood loss. Arterial blood pressure (ABP) and heart rate were recorded at the baseline and then after spinal block every one minute for three minutes then every 3 min till the delivery of the fetus because our hypothesis was that PLR would improve ABP in the early intraoperative period before delivery of the fetus and before oxytocin infusion.

### Statistical analysis

Power analysis was done on incidence of PSH after spinal block as this is the primary outcome of our study. Previous studies reported an incidence of 60% for PSH in obstetric patients. Sample size was calculated to detect a 50% decrease in the incidence of PSH. Taking a study power of 95% and *P* value less than 0.05 a minimum number of 75 patients were required for each group after exclusion of dropouts. Continuous data was presented as means (standard deviations) and medians (quartiles) and analyzed using unpaired *t*-test and Wilcoxon rank test as appropriate. Categorical data was presented as frequency (%) and analyzed using Chi-square test. Repeated measures were analyzed using two way Analysis of variance (ANOVA). *P* value less than 0.05 was considered statistically significant.

## Results

**Fig. 1:**
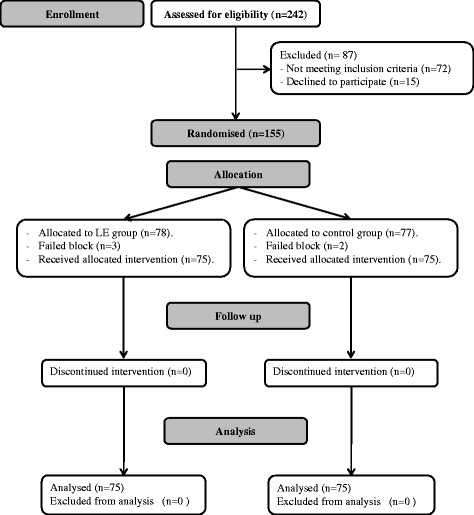
Flow chart for patient enrolment. LE: Leg elevation

**Fig. 2:**
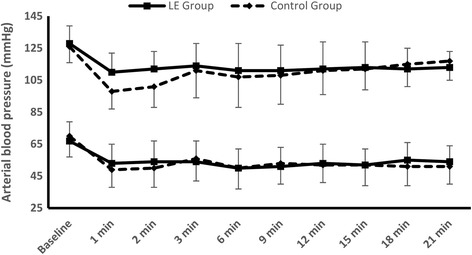
Systolic and diastolic blood pressure. Data are means, error bars are standard deviations. LE: Leg elevation. *denotes statistical significance between the two groups

**Fig. 3:**
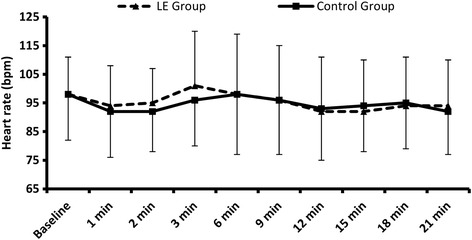
Heart rate. Data are means, error bars are standard deviations. LE: Leg elevation

Two hundred and forty two parturients were assessed for eligibility, 155 parturients were meeting inclusion criteria. Five parturients were excluded due to failed spinal block and finally 150 parturients were available for analysis (Fig. 1). Demographic data (age, weight, height, and obstetric data) were comparable between both study and control groups (Table 1). LE group showed a lower incidence of PSH and lower ephedrine consumption compared to control group (34.7% Vs 58.7%, *P* = 0.005), (4.9 ± 7.8 mg Vs 10 ± 11 mg, *P* = 0.001) respectively (Table 1). Odds ratio for development of PSH with LE was 0.374 with 95% confidence interval (0.193-0.724). There was a significant decrease in systolic and diastolic blood pressure readings in both study and control groups after spinal block compared to the baseline values. LE group showed higher systolic and diastolic blood pressures compared to the control group after 1 min and 2 min from spinal block (Fig. 2). There was no significant difference between both groups regarding heart rate, blood loss, urine output, intraoperative and postoperative nausea and vomiting (Table 1; Fig. 3).

**Table 1:** Demographic data and patients’ outcomes

	LE group (*n* = 75)	Control group (*n* = 75)	*P* value
Age (years)	29 ± 4	30 ± 4	0.13
Weight (Kg)	69 ± 7	72 ± 8	0.02*
Time from SAB to delivery of the foetus (minutes)	15.4 ± 6.3	15.9 ± 6.8	0.64
Total infused volume (mL)	1790 ± 408	1865 ± 450	0.29
Urine output (mL)	570 ± 90	590 ± 69	0.12
Blood loss (mL)	772 ± 165	820 ± 190	0.1
Incidence of hypotension	26(34.7%)	44(58.7%)	0.005*
Ephedrine consumption (mg)	4.9 ± 7.8	10 ± 11	0.001*
Nausea & vomiting	8(10.7%)	14(18.7%)	0.24
Incidence of bradycardia	4(5.3%)	7(9.3%)	0.53
Hypotensive episodes			0.014*
One	17(22%)	23(30%)	
Two	8(10%)	15(20%)	
Three	1(1.3%)	6(8%)	

*LE* leg elevation, *SAB* subarachnoid block, *NS* not significant

*denotes statistical significance (*P* value < 0.05)

Data are presented as mean ± standard deviation and frequency (%)

## Discussion

We reported a significant decrease in the incidence of hypotension and intraoperative vasopressor requirements in the LE group compared to the control group. LE decreased the incidence of hypotension by 40.9%.

Spinal block results in thoracolumbar sympathetic fiber block leading to profound vasodilatation [1]. Vasodilation decreases arterial blood pressure; moreover, it decreases venous return and consequently accentuates the hypotension [2]. LE induces auto-transfusion of blood from the lower extremities to the central circulation; thus, LE increases the cardiac preload and consequently the cardiac output [5, 6]. A previous study using radiolabeled erythrocytes reported a reduction of 34 ± 4% in counts from the radiolabeled intravascular space from the calves following LE [8] that is corresponding to about 150 mL [6]. Although the volume transported during leg elevation is not large, we assume that it is quite effective in decreasing the incidence of PSH for two reasons: 1-Being transfused in a very short period. 2- Being blood and not ordinary fluids.

Our results differed from Rout et al. [9] findings who reported no advantage for patients’ leg elevation on the incidence of hypotension. Rout et al. included only 31 patients in each group so their study could be underpowered to prove this hypothesis. Our study has the advantage of the conservative assumption in sample size calculation with a study power 95%. A recent study investigated the ability of passive leg raising (which is a maneuver near to our maneuver) in preoperative prediction of PSH; however, passive leg raising test was not effective for this aim [10].

Although the incidence of hypotension was lower with LE group, most of the serial ABP readings after 3 min were comparable between both groups (Fig. 2); this is mainly due to the abrupt administration of vasopressors in patients with hypotension. It is to be noted that ephedrine consumption was significantly lower in LE group.

Many protocols that target increasing central blood volume have been investigated for abolishing the incidence of PSH during CS. Protocols for increasing central blood volume included patient positioning protocols, leg wrapping, and sequential compression devices. In a qualitative systematic review, Morgan et al. [11] reported that leg wrapping and thromboembolic stockings decreased but did not eliminate the occurrence of PSH during CS. However, Morgan et al. did not report a clear evidence for the value of leg elevation in prevention of PSH. Many positioning protocols have been investigated since then such as delayed supine positioning [12], left lateral tilting [4], lateral positioning during [13] and after [14] spinal block, head up and head down position [4] after spinal block; however, the latest Cochrane database reviews [3, 4] did not favor any positioning protocol over the other.

CS is an operation performed in every hospital; thus, a proper protocol for prophylaxis from PSH during CS should be simply applied by physicians with moderate experience. Proper protocol also should avoid the use of expensive and sophisticated device to be suitable for setting with limited resources. Our findings give a simple, rapid, and effective method for prevention of spinal hypotension without affecting the level of spinal block. In our study LE had a moderate effect that produced a significant but not huge reduction in the incidence of PSH. LE would be useful in settings with low resources to avoid excessive use of ephedrine. LE would also be useful in prevention and management in situations of profound PSH especially when combined with other measures.

Our study had some limitations; hemodynamic assessment of our patients was based only on heart rate and blood pressure. We assume that the use of advanced cardiac output monitors in future studies might be more informative for the precise effect of LE on maternal hemodynamics. We measured blood pressure at one minute intervals for three measures followed by three-minute intervals till delivery of the fetus; this might be a potential limitation because most studies reported this measure more frequently. We hypothesized that the effect of leg elevation would be at its maximum at the first 5 min. We did not combine LE with any other positioning protocol such as left uterine displacement to avoid any confounder that might affect our intervention.

Another limitation was the use of crystalloid preloading protocol although many recent recommendations suggested the use of crystalloid co-loading, colloid pre-loading, or colloid co-loading regimens [1, 15, 16]. It is to be noted that the incidence of PSH is high with most of fluid loading protocols [3]. Also, most of the recent protocols recommend phenylephrine as the drug of choice in management of PSH instead ephedrine was used in this study as it is the available vasopressor in our institute [1]. Although this might limit the value of our findings in settings with available phenylephrine and colloid fluid loading protocols, we note that the incidence of PSH is still high with most of the available prophylactic measures; thus, combination of different measures is still needed to reach a clinical satisfactory level. We recommend in future studies combination of LE with other pharmacological and non-pharmacological approaches (e.g., prophylactic vasopressors and left uterine displacement) for more efficient elimination of PSH during CS.

## Conclusion

LE performed immediately after spinal block reduced the incidence of PSH in parturient undergoing CS.

## Abbreviations

ABP: Arterial blood pressure
ANOVA: Analysis of variance
ASA: American Society of anesthesiologists
CO: Cardiac output
CS: Cesarean section
ECG: Electrocardiograph
LE: Leg elevation
PSH: Post-spinal hypotension
SBP: Systolic blood pressure
SV: Stroke volume
